# Supplementary material for: Effect of Coffee on the Bioavailability of Sterols
Source: Foods. 2022 Sep 20;11(19):2935. doi: 10.3390/foods11192935 (PMC9563500; doi:10.3390/foods11192935)
Supplement: Supplementary file 1 [file foods-11-02935-s001.zip › foods-1868043-supplementary.pdf]

foods

Supplementary material for

Effect of Coffee on the Bioavailability of Sterols

Cristiana L. Pires [1,2], Inês M. V. Silva [1], Manuel A. Coimbra [3], Maria João Moreno [1,2,*] and Filipe Coreta-Gomes [3,*]

Section **Page**

S1 - Analytical procedure for the quantification of Lucifer yellow S1
Figure S1—Graphical representation of the LY calibration curves. S1
Table S1—Parameters obtained for the LY calibration curves. S2

S2 - Analytical procedure for the quantification of DHE S3
Figure S2—Graphical representation of the LY calibration curves. S3
Figure S3—Graphical representation of the LY calibration curves. S4
Table S2—Parameters obtained for the LY calibration curves S4

S3—Effect of sample complexity and pre-treatment in the recovery of LY and DHE S5
Table S3—% recovery of LY and DHE S5

S4—Cytotoxicity of bile salts and coffee samples S6
Figure S4—Cytotoxicity of GDCA, GCA and GDCA/GCDCA/GCA mixtures. S6
Figure S5—Cytotoxicity of coffee samples. S7

S5—Solubility of DHE in GCA micelles S8
Figure S6—Concentration dependence of DHE fluorescence intensity. S8

S6—Calculation of the amount of lipids in the plasma membrane of Caco-2 monolayers S9

S7—Contribution of coffee to the sample fluorescence in the UV S10
Figure S7—Confocal microscopy image of coffee D1 S10

S1 - Analytical procedure for the quantification of Lucifer yellow

The DHE permeability assays were performed in the presence of LY to evaluate if the concentrations of coffee samples and bile salt GCA result in effects on the tightness of the cell monolayer. This control was justified although no cytotoxicity was observed when evaluated by the MTT test. This because small changes in the cell condition may lead to large effects in the integrity of the cell monolayer and result in a significantly higher permeability for slowly permeating solutes. The samples analyzed contained both DHE and LY fluorescent probes. Their spectroscopic properties are however different, with DHE absorbing light and emitting fluorescence in the UV region while LY absorbs light in the blue region of the spectrum and emits fluorescence in the green. No interference of DHE in LY fluorescence intensity is therefore observed, allowing the direct quantification of LY in the collected sample. LY fluorescence intensity was measured in a SpectraMax iD5 Multi-Mode Microplate Reader (Molecular Devices Corporation Sunnyvale, CA, USA) at excitation and emission wavelengths of 435/560 nm. Two sets of LY solutions where used for the calibration; one with LY dissolved in HBSS and the other in HBSS with 100 µM of BSA, corresponding respectively to the transport media used in the apical and basolateral compartments of the permeability assays. Each calibration curve was built from 3 independent set of solutions, with the average results shown in **Figure S1**, and the corresponding parameters presented in **Table S1**.

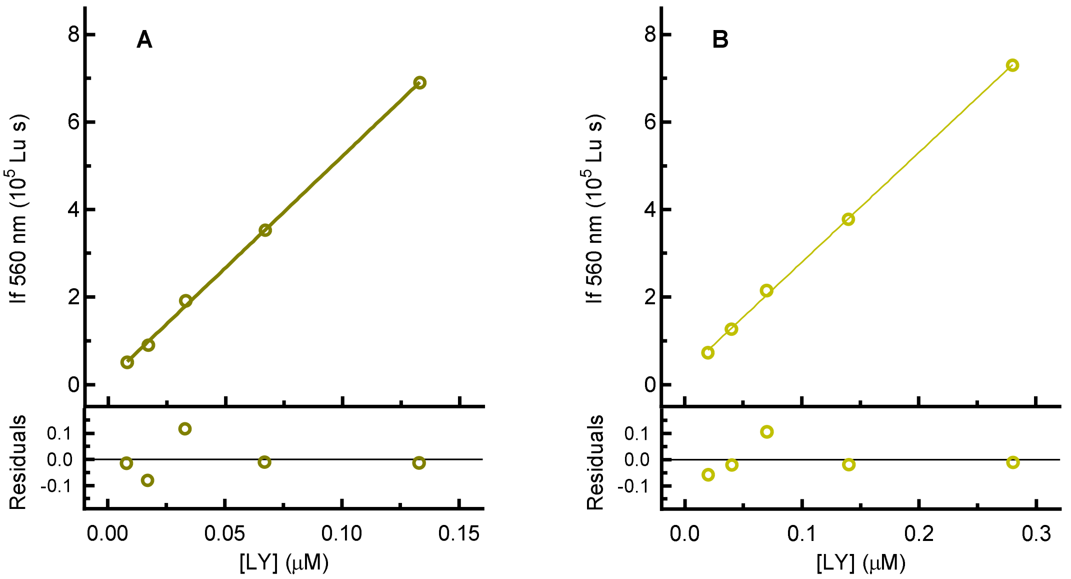

Figure S1. Final calibration curves for the quantification of LY in samples from the apical (in HBSS, plot A) and the basolateral (in HBSS +BSA 100 µM, plot B) compartments. Three independent samples were analyzed for each concentration, the data presented corresponds to the average of the fluorescence emission at 560 nm when excited at 435 nm. The standard deviation associated with the fluorescence was close to 15 % of the signal for all conditions and LY concentrations tested. The lines correspond to the best fit of a straight line with the parameters presented in Table S1. The lower insets are the best fit residuals. The parameters obtained for the calibration curves are provided in Table S1.

Table S1. Parameters of the calibration curves obtained for the quantification of LY concentrations in samples from the apical (A) and basolateral (B) compartments in the permeability assay.

**Calibration Curve**	**Solvent**	**Linearity**		**MDL (μM)** [a]	**MQL (μM)** [b]
		r^2	Calibration range (μM)		
A	HBSS	0.9997	0.01 − 0.13	0.005	0.014
B	HBSS+BSA 100 μM	0.9997	0.02 − 0.28	0.009	0.026

[a] method detection limit (MDL) calculated as $3\times S_{y/x}/b$ where $S_{y/x}$ is the standard error of the estimated curve and b is the slope of the calibration curve. [b] method quantitation limit (MQL) calculated as $10\times S_{y/x}/b$.

S2

S2 - Analytical procedure for the quantification of DHE

The quantification of the DHE in the samples collected from the Caco-2 permeability assays was performed by reverse phase HPLC using an isocratic elution with 100 % methanol, a Zorbax ODS C18 (250×4.6 mm, 5 μm) column with a pre-column with the same stationary phase and 12.5 mm length, and fluorescence detection at λex: 324 nm and λem: 372 nm. The chromatograms obtained for the samples in the apical compartment at the beginning and end of the permeability assay (black and dashed dark grey lines respectively) and in the basolateral compartment at the end of the assay (light grey lines) are presented in **Figure S2**, for DHE alone (plot **A**) and in the presence of coffee (plot **B**). The comparison of the chromatograms shows that DHE elution profile was not affected by the presence of the coffee sample nor by the 6 h incubation or transport through the Caco-2 monolayer. The DHE retention time was around 12 min, equivalent to that obtained for the DHE solutions in methanol used to establish the calibration curves. The chromatograms were integrated from 12.5 to 16.5 min, considering a linear baseline. The calibration curves are shown in **Figure S3** for both low and high DHE concentrations (plots A and B respectively). The overall results obtained for the performance of the analytical method are presented in **Table S2**.

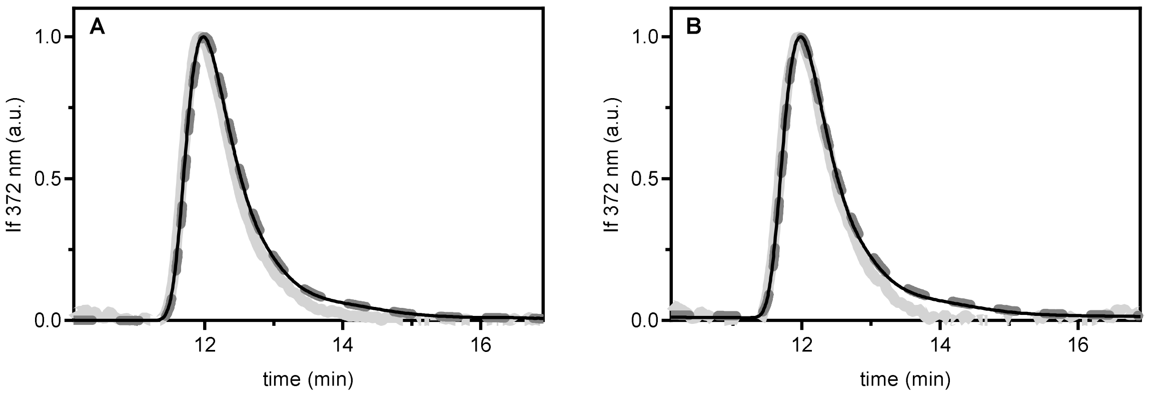

Figure S2. Chromatograms of the fluorescence emission at 372 nm when excited at 324 nm obtained from permeability assays through Caco-2 monolayers with DHE alone (A) or with DHE and coffee D1 diluted 16x sample (B). The samples where taken from the apical compartment at the beginning of a permeability assay (black line), from the apical compartment after incubation with the cell monolayer for 6 h (dark grey line) and in the basolateral compartments (light grey line).

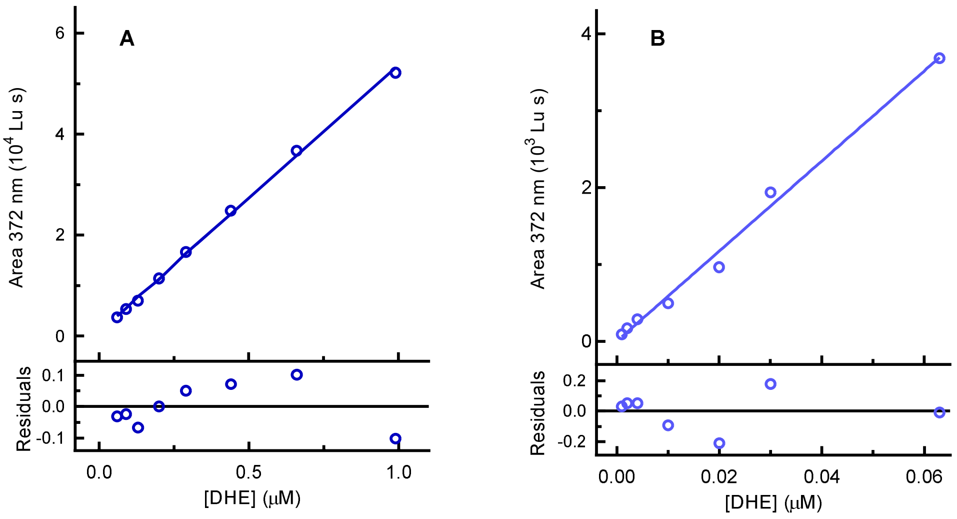

Figure S3. Final calibration curves for the quantification of DHE for the samples collected from the apical (A) and basolateral (B) compartments. The areas correspond to integration between 12.5 and 16.5 min of the fluorescence emission at 372 nm when excited at 324 nm. Three independent samples were analyzed for each concentration, the data presented corresponds to the average with a standard deviation of about 5% and 11% for plot A and B, respectively. The lines correspond to the best fit of a straight line with the parameters presented in Table S2. The lower insets are the best fit residuals. The parameters obtained for the calibration curves are provided in Table S2.

Table S2. Parameters of the calibration curves obtained for the quantification of DHE concentrations in samples from the apical (A) and basolateral (B) compartments in the permeability assay.

**Calibration Curve**	**Linearity**		**MDL (μM)** [a]	**MQL (μM)** [b]
	r^2	**Calibration range (μM)**		
A	0.9992	0.06 – 0.10	0.046	0.14
B	0.9997	0.001 – 0.063	0.0017	0.0053

[a] method detection limit (MDL) calculated as $3\times S_{y/x}/b$ where $S_{y/x}$ is the standard error of the estimated curve and b is the slope of the calibration curve. [b] method quantitation limit (MQL) calculated as $10\times S_{y/x}/b$.

S3 – Effect of sample complexity and pre-treatment in the recovery of LY and DHE

Due to the presence of coffee fibers or BSA in the samples collected from the permeability assay, the samples could not be directly used for the quantification of DHE by HPLC. As described in the main manuscript, the samples were first lyophilized, followed by DHE solubilization in water:methanol 1:7 (v/v). The efficiency in DHE recovery was evaluated using solutions containing the relevant sample components and well-defined DHE concentrations. The results obtained are shown in **Table S3**.

The effect of DHE/GCA and coffee sample on the quantification of LY by fluorescence using the plate reader was also evaluated, and is also shown in **Table S3**.

Table S3. % recovery of LY and DHE, see text above for details.

Analyte	Medium composition (in HBSS)	Analyte concentration (μM)	Recovery (%)
			Mean ± SD
LY	GCA 10 mM + 1% (v/v) DMSO + coffee sample diluted 16×	20	97.7
DHE[a]	BSA 100 μM + 1% (v/v) DMSO	1	97.6 ± 2.3
	GCA 10 mM + 1% (v/v) DMSO + coffee sample diluted 16×	10	97.7 ± 8.4

[a] (*n* = 3 for each condition).

S4 – Cytotoxicity of bile salts and coffee samples

Cytotoxicity studies were conducted to guide the selection of the composition and concentration of bile salt solution used to mimic the intestinal lumen in the permeability assays. The toxicity of GDCA, GCA and their mixtures with GCDCA is shown in **Figure S4** for different bile salt concentrations. With the exception of GCA solutions at concentrations ≤ 12.5 mM, all bile salt solutions showed high toxicity to Caco-2 cells after incubation for 2 h.

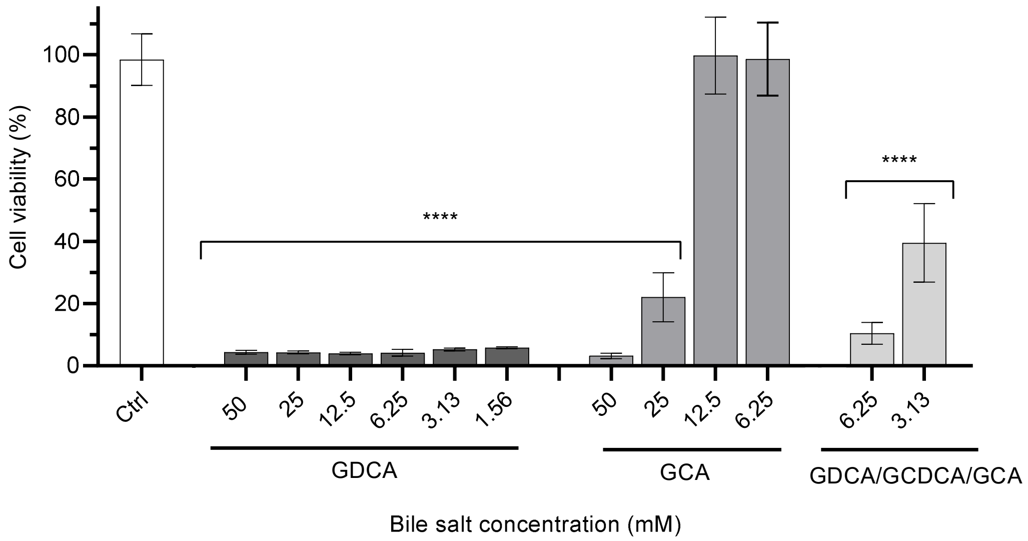

Figure S4. Evaluation of Caco-2 viability after incubation for 2 h with distinct concentrations and compositions of bile salt solutions. Cell viability was assessed using the MTT assay. The bile salt solutions were prepared in HBSS and control cells were incubated in HBSS only (100 % viability). Statistically significant differences between the negative control are represented as **** $p < 0.0001$.

The effects of coffee samples on Caco-2 viability were also characterized, **Figure S5**. At low coffee concentrations (dilution of 32 times relative to an expresso like coffee), no cytotoxicity was observed. However, at high coffee concentrations (dilutions of 4 and 8 times relative to an expresso like coffee) the presence of the coffee sample leads to a moderate toxicity, with cell viability decreasing to 50 % for some coffee samples. The results obtained at intermediate dilutions are dependent on the coffee sample.

The bars on the right of each plot show the cytotoxicity of solutions containing both coffee and the bile salt GCA. It is observed that the toxicity of all coffee samples is significantly decreased, with a cell viability close to 100 % at least up to a coffee concentration corresponding to 8 times dilution.

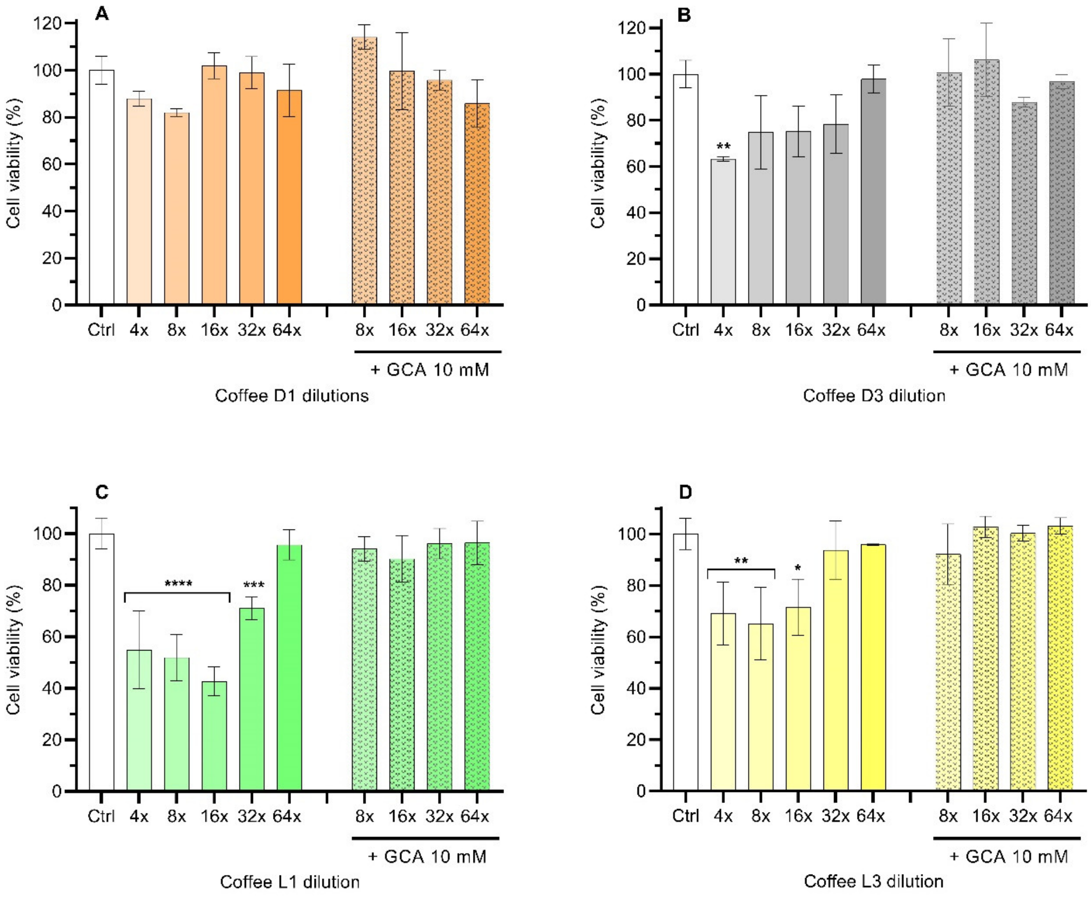

Figure S5. Effect of coffees sample processing, light (L) and dark (D) roasts, fine (1) and coarse (3) grindings, on the viability of Caco-2 cells. Dilutions were prepared with HBSS or GCA 10 mM in HBSS. Statistically significant differences between the negative control are represented as **** $p < 0.0001$, *** $p < 0.001$, ** $p < 0.01$, and * $p<0.05$. See caption of Figure S4 for additional details.

S5 – Solubility of DHE in GCA micelles

The solubility of DHE in the GCA micelles at the concentration of GCA used in the permeability assays (10 mM) was characterized from variations in DHE fluorescence intensity. The concentration of DHE was varied from 1 to 70 μM, with DHE being added to the GCA solution by squirting a small volume of a DHE solution in DMSO (% DMSO < 1 %). The solutions obtained were optically clear and stable. However, the fluorescence intensity measured in a right-angle geometry was not linearly dependent on the intensity of light absorbed, **Figure S6**. This behaviour is in part due to geometrical constraints due to the right-angle detection, with a significant fluorescence being emitted but not detected. In addition, it shows that some of the DHE is not solubilized by the GCA micelles. The stability of the samples suggests that under those conditions the non-solubilized DHE is present as small aggregates.

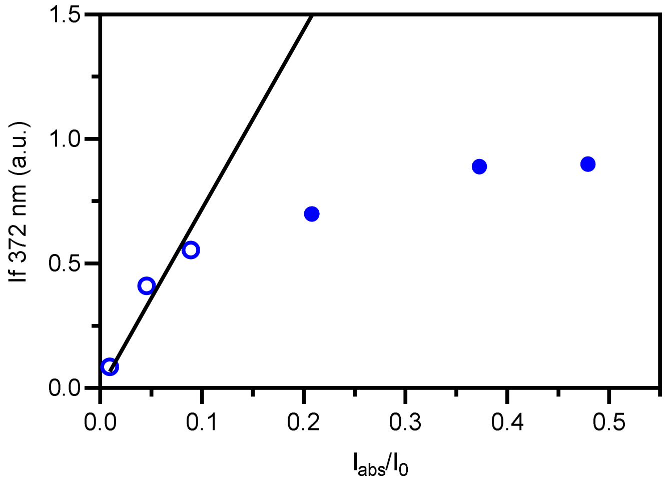

Figure S6. Dependence of DHE fluorescence intensity ◎ex/◎em = 324/372 nm on the intensity of light absorbed by DHE, when added to aqueous solutions containing 10 mM GCA. The hollow symbols correspond to DHE concentrations equal to 1, 5 and 10 μM, and the filled symbols correspond to DHE at 25, 50 and 70 μM. The line represents the best fit for the hollow symbols.

S6 – Calculation of the amount of lipids in the plasma membrane of Caco-2 monolayers

For the calculation of the total surface of plasma membrane in the Caco-2 monolayer, cells were modelled as rectangular cuboids with 20 μm in the x and y direction and 8 μm thickness.

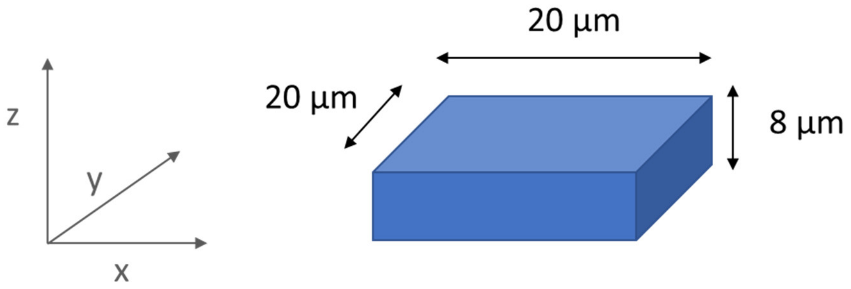

If the cells are considered flat, the area of the apical surface (a_A) is equal to a_A=20×20=400 μm^2 for each cell, and the total area of the plasma membrane (a_{Cell}) is equal to a_{Cell}=2×20×20+4×8×20=1440 μm^2. The number of cells (N_{cell}) required to cover the whole surface of the insert (S=1.12 cm^2) is given by S/a_A= N_{cell}=2.8×10^5, leading to a total surface of plasma membrane (S_{Cell}), S_{Cell}= N_{cell}× a_{Cell}=4×10^8 μm^2. Considering an average area of lipid (a_L), a_L=0.5 nm^2 per lipid (a good approximation for cholesterol enriched membranes), the total number of lipid molecules in the plasma membrane of the Caco-2 monolayer is equal to S_{cell}/a_L=8×10^{14} lipid molecules. Division by the Avogadro number gives the moles of lipids (n_L) in the plasma membrane of the cell monolayer, n_L=1.34 nmol.

A major approximation in the above calculations is the assumption that the apical surface of the cell plasma membrane is flat. This membrane is known to have villi and microvilli which increases significantly the membrane surface area. The total moles of lipid in the apical portion of the plasma membrane may be expressively larger, with a 10-fold increase being considered in the calculations given below. The total surface of each cell would thus be a_{Cell}=4000+400+4×8×20=5040 μm^2 (apical+basal+lateral), leading to a total surface of plasma membrane in the cell monolayer equal to S_{Cell}=1.4×10^9 μm^2, corresponding to approximately 5 nmol lipid.

S7 – Contribution of coffee to the sample fluorescence in the UV

The confocal image of a coffee sample deposited in the inserts used for the permeability assays is shown in **Figure S7**. This control was required to evaluate the possible contribution of the coffee components to the significant increase in the fluorescence intensity observed by confocal microscopy for the samples containing coffee and DHE. The control with the fluorescence image of the insert membrane only is also shown.

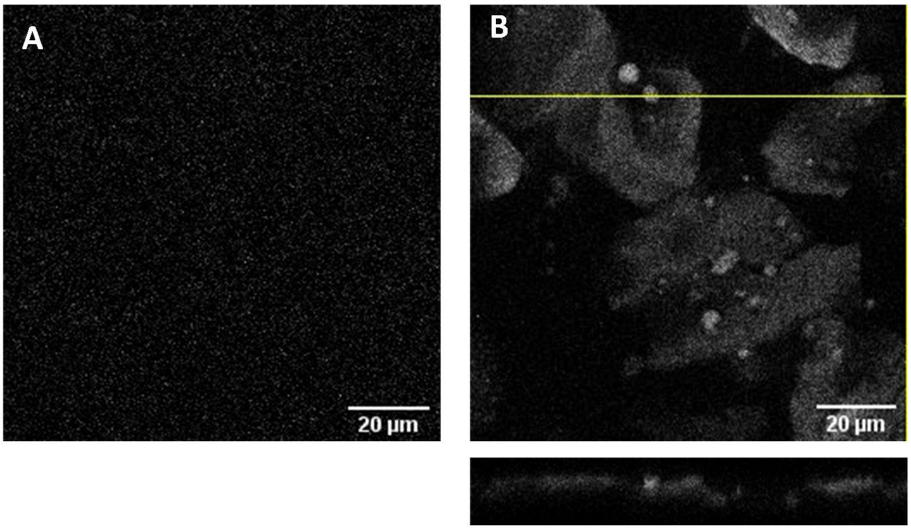

Figure S7. Confocal microscopy images of the coffee D1 obtained with excitation light at 405 nm and emission detection from 408-490 nm. Representative image of a cross section of a z-x plane are shown for the coffee solution (B), corresponding to the position of the yellow line for coffee sample (B).
